# Targeting vascular senescence in cardiovascular disease with aging

**DOI:** 10.20517/jca.2023.45

**Published:** 2024-02-28

**Authors:** Shelby A. Hall, Lisa A. Lesniewski

**Affiliations:** 1 Department of Nutrition and Integrated Physiology, University of Utah, Salt Lake City, UT 84112, USA.; 2 Internal Medicine, University of Utah, Salt Lake City, UT 84112, USA.; 3 Geriatric Research Education and Clinical Centers, Veterans Affairs Medical Center, Salt Lake City, UT 84148, USA.; 4 Nora Eccles Harrison Cardiovascular Research and Training Institute, University of Utah, Salt Lake City, UT 84112, USA.

**Keywords:** Vascular aging, endothelial dysfunction, senescence, senolytics, cardiovascular disease

## Abstract

Aging is a major risk factor for atherosclerosis and cardiovascular disease (CVD). Two major age-associated arterial phenotypes, endothelial dysfunction and large elastic arterial stiffness, are autonomous predictors of future CVD diagnosis and contribute to the progression of CVD in older adults. Senescent cells lose the capacity to proliferate but remain metabolically active and secrete inflammatory factors termed senescence-associated secretory phenotype (SASP), leading to an increase in inflammation and oxidative stress. Accumulation of senescent cells is linked with the progression of age-related diseases and has been known to play a role in cardiovascular disease. In this brief review, we describe the characteristics and mechanisms of senescent cell accumulation and how senescent cells promote endothelial dysfunction and arterial stiffness. We focus on a range of novel therapeutic strategies aimed at reducing the burden of endothelial dysfunction leading to atherosclerosis through targeting senescent cells. Studies have begun to investigate a specific class of drugs that are able to selectively eliminate senescent cells, termed senolytics, which have shown great promise in reversing the aging phenotype and ameliorating pathologies in age-related disorders, creating a new opportunity for aging research. Generating therapies targeting the elimination of senescent cells would improve health span and increase longevity, making senolytics a promising therapy for cardiovascular diseases.

## Graphical Abstract

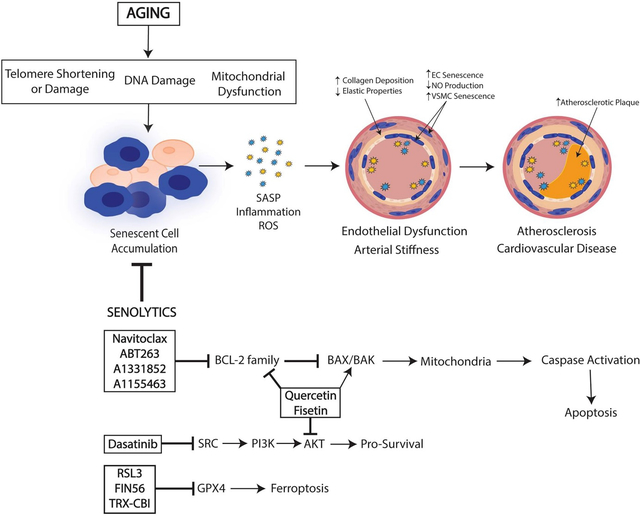

## INTRODUCTION

### Cellular senescence is a hallmark of aging and cardiovascular dysfunction

Cardiovascular disease (CVD) is the leading cause of death worldwide. In America, the cost of cardiovascular disease in the healthcare system has reached over $300 billion per year^[1]^. Aging is associated with a progressive decline in various physiological processes, leading to increased risk for multiple chronic diseases. Indeed, aging is a major risk factor for CVD, with over 50% of all deaths in Americans aged over 85 due to CVDs^[1,2]^. Thus, age-related CVD is an important area of study and understanding why age is such a critical component of disease etiology is critical. Although there are many studies relating CVD to aging, the molecular mechanisms underlying the age-associated increase in CVD are still being elucidated and therapies targeting the age-related mechanisms of cardiovascular disease may prove efficacious. Aging has a remarkable impact on the heart and arterial system, leading to cardiac hypertrophy^[3]^, altered left ventricular (LV) diastolic function, increased arterial stiffness and thickening^[4]^, and impaired endothelial function^[5–7]^. These pathophysiological changes contribute to the increased prevalence of CVDs such as atherosclerosis, hypertension, myocardial infarction, and stroke^[8]^. Endothelial cells (ECs) line the inside of all blood vessels and are more prominent in the vasculature than any other cardiac cell type, with a probable ratio of ECs to cardiomyocytes being 3:1^[9,10]^. This indicates an essential role for endothelial cells in cardiovascular health and disease progression. Indeed, ECs participate in many biological processes such as arterial stiffness, angiogenesis, coagulation, and systemic metabolism^[11,12]^, and maintaining EC homeostasis is critical for preventing endothelial dysfunction, atherosclerosis, and CVD.

Cellular senescence, the arrest of cell division in response to the activation of tumor suppressors, contributes to aging and age-related diseases. Senescence is a response mechanism to stress activated by a variety of stimuli, including telomere attrition, hypoxia, oxidative stress, mitochondrial dysfunction, and impaired autophagy^[13]^. There are two main types of cellular senescence: replicative senescence and stress-induced premature cellular senescence. Replicative senescence occurs through telomere attrition, where telomeres positioned at the end of a chromosome replicate incompletely during cell division. Telomeres play a large role in chromosomal stability and DNA replication, and when telomeres shorten, it leads to DNA damage and induces replicative senescence, mainly through the p53 or p16Ink4a signaling pathways. Stress-induced premature senescence advances through oxidative stress or irradiation, constitutive activation of mitogenic stimuli, oncogenic activation, and metabolic stress. Stress-induced senescence is also facilitated through p53 or p16Ink4a signaling pathways. P53 signaling is predominantly activated through DNA damage and telomere dysfunction, whereas p16Ink4a signaling is mainly associated with mitogenic stress, and overall cellular stress^[14,15]^.

In response to a loss of function, senescence can act as a protective mechanism and can lead to beneficial effects on physiological and pathological processes, comprising wound healing, host immunity, and tumor suppression^[13]^. However, senescent cells can also have negative effects that are associated with a decline in overall physiological function and health. Senescence causes the loss of repair mechanisms due to cell cycle arrest. At the same time, senescent cells remain metabolically active and produce inflammatory factors, collectively termed senescence-associated secretory phenotype (SASP)^[16,17]^. With advancing age, cells chronically accumulate damage and can reach a threshold of cellular stress that promotes their withdrawal from the cell cycle, increasing senescent cell accumulation^[17]^. The discovery of replicative senescence by Hayflick and Moorfield in 1961 led researchers to believe that aging and senescence are causally linked^[18]^. In a study evaluating SASP as a potential driver of age-related dysfunction, a cohort aged 60–90 years old were assessed. SASP proteins composed of growth differentiation factor 15 (GDF15), TNF receptor superfamily member 6 (FAS), osteopontin (OPN), TNF receptor 1 (TNFR1), ACTIVIN A, chemokine (C-C motif) ligand 3 (CCL3), and IL-15 were positively associated with age, frailty, and adverse surgery outcomes. The combination of these SASP proteins expected unfavorable outcomes significantly better than a single SASP protein or age alone^[19]^. Because of the potential of senescent cells to contribute to many aging- and disease-related processes, eliminating senescent cells and attenuating SASP have emerged as possible therapeutic approaches to treat age-related diseases. However, understanding the role of senescence in the endothelium, how this contributes to CVD with aging, and whether senescence in this cell type can be pharmacologically targeted to alleviate CVD requires elucidation.

Drugs that target senescent cells are termed senolytics. The first of their types was identified using a hypothesis-driven, mechanism-based drug discovery approach. Because it was hypothesized that cell survival would be beneficial, these initial senolytics targeted anti-apoptotic, pro-survival pathways^[20]^. As proteomic and transcriptomic datasets revealed that anti-apoptotic pathways were upregulated in one or more senescent cell populations, these pathways became the target for identification of potential senolytic agents^[21,22]^. The first senolytic agents that were identified for further investigation were ones that targeted several senescent cell anti-apoptotic pathways (SCAPs), could be administered orally, and were natural products with known safety protocols to facilitate translation from bench to bedside. Dasatinib (D), the SRC/tyrosine kinase inhibitor, was one of the first senolytic agents to be intensively researched, as it has been extensively used since 2006^[23]^. In combination with dasatinib, the naturally occurring flavonoids quercetin and fisetin, which are present in fruits and other foods, have shown promise in attenuating senescent burden^[24]^. As this field progresses, key priorities for senolytic research should be the continued identification of both reliable and sensitive biomarkers for senescence, and safe, feasible, and tolerable senolytic agents that can be successfully put into the clinical setting. Here, we present a framework describing the role of senescent cells in the cardiovascular system and in the development of atherosclerosis with aging, focusing on new senolytic approaches to target senescent cells and treat disease.

## AGING AND SENESCENCE

### Hallmarks of cellular senescence

Cell cycle arrest is a key characteristic of the senescent phenotype, promoted by the activation of p53/p21^WAF1/Cip1^ and the Rb-p16^INK4A^ axes^[13]^. DNA damage, including irreparable double-stranded breaks, is a common trigger of cell cycle arrest that leads to the activation of the DNA damage response (DDR) pathway^[13]^. Senescence entry can occur via p53, a tumor suppressor pathway induced in response to DNA damage^[15]^. P53 provokes the senescence response by increasing the expression of the p21 cyclin-dependent kinase inhibitor (CDKI), leading to cell cycle arrest^[25]^. P21, in turn, inhibits the phosphorylation and inactivation of the tumor suppressor pRB, which can also be controlled by another CDKI, p16, which has also been reported to be upregulated in senescent cells^[15]^. Both p21 and p16 are common markers of senescence. DNA damage such as cytoplasmic chromatin fragments and mitochondrial DNA damage are also evident in senescent cells^[13]^. Telomeric changes, such as telomere shortening and dysfunction, are also associated with senescence. When DNA machinery lacks the ability to replicate telomeric DNA completely, the shortening of telomeres occurs. Therefore, telomerase expression or recombination of telomeric DNA may become impaired, leading to telomere attrition after each round of cell division^[26,27]^. Telomere dysfunction is identified by telomere dysfunction-induced foci (TIF), i.e., increases in p53 localized to the telomere, which indicate telomere-associated DNA damage.

Another marker of senescence includes the forkhead box O (FOXO) family of proteins is made up of 4 transcription factors sharing the forkhead box domain (FH). FOXOs are tumor suppressors that contribute to the regulation of many functions including cell growth and survival, metabolism, and oxidative stress by regulating the expression of several target genes^[28,29]^. Oncogenic signaling has been shown to activate cellular senescence as a protective mechanism against cancer; this method has been termed oncogene-induced senescence (OIS)^[29]^. Both FOXO4 and p53-mediated transcriptional activation of p21 triggers cellular senescence. Research has shown that inducing senescence *in vitro* increases the expression of FOXO4, with no changes to the other FOXO proteins, signifying an explicit function for FOXO4 in the progression of cellular senescence. Baar *et al*. demonstrated that FOXO4 induces senescence and preserves the viability of senescent cells by suppressing their apoptotic responses. FOXO4 can directly bind to and activate the p53-dependent transcription of the senescence-associated p21 gene^[30]^. Although the specific mechanisms of FOXO4-dependent p53 activation of p21 transcription are unknown and require more research, the FOXO-p53 axis is a key marker of cellular senescence and may represent a potential therapeutic approach in targeting senescence in age-related diseases.

When a cell enters senescence, it can rewire its metabolic activity, exhibit an enlarged flattened morphology, and show increased β-galactosidase (β-gal) activity, a widely accepted hallmark of senescence^[13,31]^. Senescence-associated β-gal activity has been shown to be increased in atherosclerotic regions and prominent in vascular and arterial dysfunction^[11]^. Although senescent cells are no longer able to proliferate or divide, they are still able to produce an array of SASP factors (interleukin-6, interleukin-8, monocyte chemoattractant protein-1, plasminogen-activated inhibitor-1, and many others), contributing to inflammation, dysfunction, and disease^[32]^. When SASP is chronic, it can trigger paracrine senescence in nearby healthy cells both in culture and in human and mouse models of oncogene-induced senescence^[33,34]^. Among the primary SASP drivers are the transcription factors, NF-kB, C/EPBbeta, and GATA4; the signaling pathways, mammalian target of rapamycin (mTOR) and p38 mitogen-activated protein kinase (p38MAPK)^[35]^; and DNA sensors, cGMP-AMP (cGAMP) synthase (cGAS). NF-kB, C/EPBbeta, and GATA4 can activate p53, p21, and p16 signaling pathways in response to DDR (DNA Damage Response), arresting the progression of the cell cycle, inducing cellular senescence and increasing SASP production^[36]^. Senescent cells can also activate the mTOR pathway, which plays a critical part in regulating cellular metabolism, cell growth, and autophagy in senescent-associated diseases. Sung *et al*. found senescent cells displayed increased activity of mTOR and therefore reduced levels of signal-associated autophagy proteins, and inhibition of mTOR pathway led to significant decreases in senescence and increases in signal-associated autophagy proteins^[37]^. The findings that inhibition of mTOR activity is linked predominantly to inhibition of cellular senescence and prolonged lifespan in model organisms are well described. mTOR drives senescence through the p53/p21 and p16/pRB pathways and is an important contributor to the progression of senescence and age-related diseases. cGAMP and cGAS activate the adaptor protein STING, leading to an increase of type I interferons and inflammatory cytokines, contributing to the inflammatory response^[38]^. Senescence cells display activated cGAS-STING pathway transcription levels as well as increased expression of inflammatory cytokine mRNAs. In one study, inhibition of cGAS mitigated DNA damage-induced IL-6 expression and cellular senescence in vascular cells^[39]^. In mice chronically treated with doxorubicin to induce senescence and cardiotoxicity, the inhibition of endothelial-specific STING significantly prevented doxorubicin-induced cardiotoxicity and endothelial dysfunction^[40]^. Indeed, the cGAS STING pathway may be a novel therapeutic target for eliminating senescent cells and preventing heart disease. Cell surface proteins have also been identified as SASP regulators in replicative senescence; these include notch1 and dipeptidyl-peptidase 5 (DPP4)^[41]^. It has been previously shown that senescent cells can communicate with their microenvironment through NOTCH signaling, an increase in reactive oxygen species (ROS), development of cytoplasmic bridges, and excretion of extracellular vesicles such as exosomes^[13]^, which are important characterizations of the senescent secretome. DPP4 has also recently been utilized as an *in vitro* marker of senescence^[42]^. Other newly identified markers of senescence include PD-L^[43]^, uPAR^[44]^, GPNMB^[45]^, and CD153^[46]^. The urinary marker α-Klotho was also recently identified as a non-invasive way to measure cellular senescence that may be useful in clinical trials^[47]^. However, none of these senescence-associated changes/markers are fully sensitive or specific to senescence, and as a result, elucidating the effects of aging, disease, or interventions on senescent cell accumulation or clearance requires the use of multiple markers.

Dysregulated metabolism in senescent cells is associated with dysfunctional mitochondria and impaired mitochondrial dynamics. This mitochondrial dysfunction leads to amplified production of mitochondrial reactive oxygen species (ROS) that can damage macromolecules including proteins, lipids, and DNA^[48]^. Mitochondrial dysfunction occurs with aging and contributes to vascular endothelial dysfunction, increased arterial stiffness, and cardiovascular disease through increases in mitochondrial ROS^[49]^. Likewise, mitochondrial ROS has also been shown to trigger DNA damage through the shortening of telomeres in addition to telomere-induced senescence^[50]^. Senescent cells have dysfunctional mitochondrial respiration and greater mitochondrial superoxide production compared to non-senescent cells^[51]^. Interventions known to decrease mitochondrial ROS have demonstrated retardation of telomere shortening, leading to improvements in lifespan^[48]^. Mitochondria in senescent cells become hyperfused and elongated, suggesting mitochondrial dysfunction is an indicator of senescence and a possible target for senescent cell clearance^[52]^. An elongated and hyperfused mitochondrial network is associated with a reduction in FISI expression, a protein important in mitochondrial fission. Studies have shown that the knockdown of FIS1 has led to an abundance of ROS production and the induction of cellular senescence^[53]^. However, overexpression of FIS1 prevented mitochondrial elongation and reversed the senescent phenotype^[53]^, suggesting mitochondrial fission may protect against cellular senescence. Mitoquinone (MitoQ) is a highly effective mitochondrial antioxidant. Treatment with MitoQ has been shown to improve arterial stiffness *in vivo*, and *in vitro* evidence suggests MitoQ may also delay the development of senescence^[54,55]^. Interventions aimed at reducing mitochondrial ROS with MitoQ have been shown to decelerate telomere shortening and extend replicative lifespan^[56]^. Thus, dysfunctional mitochondria are important factors in senescence accumulation and the development of CVD and other age-related diseases. Research targeting mitochondrial ROS to delay senescence, as well as the senolytic potential of MitoQ, is currently being conducted with promising results.

## CARDIOVASCULAR-SPECIFIC IMPLICATIONS OF CELLULAR SENESCENCE AND AGING

Vascular aging, a predictor of cardiovascular diseases such as hypertension, aortic aneurysms, and atherosclerosis^[57]^, is characterized by reduced endothelium-dependent dilation and increased arterial stiffness. Vascular aging is also associated with elevated systolic blood pressure, decreases in diastolic pressure, and increased central venous pressure^[4]^. These functional and structural changes are associated with endothelial cell (EC) and vascular smooth muscle cell (VSMC) dysfunction, infiltration of immune cells in arterial tissues, and alterations in the extracellular matrix (ECM) of arteries. In addition, aged ECs have reduced production of vasodilators such as NO and prostacyclin and increased production of vasoconstricting factors including endothelin and ROS. Aged VSMCs have disturbed mechanosensing capabilities and adverse changes in ECM structure that contribute to stiffer arteries through increases in collagen and loss or breaks in elastin. Together, these age-related EC and VSMC changes lead to a proinflammatory, pro-thrombotic, proliferative phenotype that contributes to increases in arterial stiffening that compromise arterial compliance and increase the risk of atherosclerosis^[58]^.

Cellular senescence is a fundamental process of cellular aging, promoting a molecular microenvironment with increased inflammation and SASP. Senescence has been described across most cell types in the CV system including cardiomyocytes, endothelial cells, fibroblasts, smooth muscle cells, immune cells, and cardiac progenitor cells. While the drivers of senescence are comparable in most cell types, including telomeric shortening, oxidative stress, mitochondrial dysfunction, and DNA damage^[59]^, the consequences of senescence across cell types can vary. Based on previous studies, senescent endothelial cells appear to be major contributors to the progression and development of CVD, making them a novel target for senolytic therapy and the focus of this review^[60]^.

### Endothelial dysfunction

Endothelial cells (EC) make up the inner lining of blood vessels and are directly exposed to endogenous signals and metabolites in the circulatory system. ECs display functional heterogeneity in structure and function. ECs are necessary components of many biological processes, such as vasoreactivity, arterial stiffness, angiogenesis, coagulation, and systemic metabolism. ECs that line the large elastic arteries have important roles in sustaining vascular homeostasis. The main function of the endothelium is to produce vasoactive substances, mainly nitric oxide (NO), to regulate vascular tone. In addition, ECs form a continuous monolayer that acts as a barrier to control substance exchange between the lumen, vascular wall, and parenchyma^[61]^. This barrier function involves the immunoregulatory recruitment of leukocytes. Upon injury or inflammatory stress, ECs become activated and present adhesive molecules including intercellular adhesion molecule-1 (ICAM-1), vascular cell adhesion molecule-1 (VCAM-1), and E-selection at their cell surface. This facilitates the migration of leukocytes to the endothelium, leading to a reactive and proinflammatory endothelial phenotype that is essential for tissue repair in response to injury. However, in a state of chronic inflammation, as with atherosclerosis or advanced age, these inflammatory responses become pathogenic and contribute to endothelial dysfunction that is associated with arterial dysfunction and disease.

Senescence plays a major role in dysfunction in the endothelium, contributing to chronic inflammation and vascular dysfunction^[62]^. The characteristics of the vascular framework, in addition to the high turnover rate of endothelial cells, likely predispose these cells to senescence. Endothelial cells are one of the primary cell types that become senescent with aging, and multiple studies have shown endothelial cells predominant in vascular beds of multiple organs and tissues with advancing age, contributing to a variety of pathological processes associated with cardiovascular disease. Senescent cells are distinct morphologically and produce a secretory phenotype specific to ECs. Senescent ECs are flat, enlarged and do not change phenotypically or in orientation in response to laminar sheer stress^[63]^. EC senescence is characterized by an age-associated reduction in EC function, including loss of control over vasodilation, blood coagulation, oxidative stress, inflammation, and immune cell infiltration^[64]^.

Senescent ECs have reduced NO production, which is the key regulator of the endothelium, an increase in ROS, p21Cip1 and p53 expression, and SASP factors, contributing to the development of inflammation and disease^[60]^. Yokoyama *et al.* demonstrated that increased endothelial p53 is associated with endothelial dysfunction. Utilizing a EC-p53KO mouse model, endothelium-dependent vasodilation and ischemia-induced angiogenesis were significantly attenuated compared to a model of p53 over-expression, making p53 an important target of endothelial function^[65]^. Senescent EC SASP is heterogeneous and determined by cell type and mechanisms of senescence. Senescent ECs can increase expression of the pro-fibrotic cytokine endothelin-1 and increase expression of proinflammatory adhesion molecules such as VCAM-1 and ICAM-1, contributing to the recruitment of immune cells to the vessel wall and surrounding tissues, further increasing inflammation^[66,67]^. Senescence accumulates in cells with aging and senescent ECs are associated with endothelial dysfunction, promoting vascular remodeling, atherosclerosis, heart failure, and other age-related diseases^[11]^. Thus, maintaining EC homeostasis via the targeting of senescent ECs is a promising approach for senolytic therapy.

### Atherosclerosis

Atherosclerosis is a chronic CVD that contributes substantial risks to human health. Atherosclerosis underlies many CVDs such as peripheral vascular disease, coronary heart disease, and stroke. Atherosclerosis develops with aging as vascular walls become stiffer, and plaques made up of cholesterol, fat, calcium, and fibrous tissue build up within blood vessels. The pathogenesis of atherosclerosis is multifaceted, involving multiple cell types, including ECs, VSMCs, foam cells, and immune cells. Endothelial dysfunction is the preliminary step in atherosclerotic progression. In addition to endothelial dysfunction, leukocyte adhesion, foam cell formation, and smooth muscle cell phenotypic transitions are key factors in the progression of atherosclerosis^[68]^. Endothelial cells can cover atherosclerotic lesions, forming a “cap” that is critical for the protection of plaque stability; however, attrition of the endothelium can lead to plaque instability at the affected lesion, which can contribute to the development of atherothrombosis^[69]^. Due to ECs’ ability to increase expression of VCAM-1 and ICAM-1 and recruit leukocytes, increased endothelial inflammation is thought to be a negative process that aids in maintaining the chronic inflammatory environment of the atherosclerotic wall. Paracrine signaling of senescent ECs to neighboring cells increases senescent cell accumulation, promoting a host of SASP factors, further contributing to disease. Senescent ECs have been shown to accumulate within atherosclerotic lesions and may contribute a causal role in the development of atherosclerosis by contributing to increased ROS and inflammatory cytokines, such as MCP-1 and Il-1B. These factors will, in turn, impair endothelium-dependent vasodilation and perturb glucose metabolism by reducing microvascular perfusion and increasing endothelial inflammation in metabolically active organs^[63]^. Maintaining endothelial cell homeostasis is important for reducing the risk of atherosclerotic progression and development.

Senescent vascular cells, including ECs, VSMCs, and foam cells, have all been observed in atherosclerotic lesions^[70]^. VSMCs are critical to atherosclerotic lesion development, and senescence accumulation in these regions may be a driving force for atherogenesis^[67]^. VSMCs can perform a phenotypic switch and acquire macrophage markers and pro-atherogenic properties^[71]^. The switching of VSMCs to macrophage-like cells may be promoted through lipid accumulation in the atherosclerotic plaque. Indeed, the increase of cholesterol in VSMCs can activate multiple proinflammatory genes and suppress VSMC marker genes, thus inducing this phenotypic shift. Senescence may also contribute to the phenotypic shift of VSMCs by increasing inflammation and SASP, although more studies are necessary. Furthermore, there is increasing evidence that endothelial and VSMC senescence-associated dysfunction promotes atherosclerotic progression by promoting enlargement of the necrotic core, increasing degeneration of the extracellular matrix (ECM), reducing cap thickness, as well as by promoting erosion, calcification, and intraplaque angiogenesis^[72]^. Loss of functional VSMC after induction of senescence can promote necrotic core formation and the inefficient clearance of senescent VSMCs leads to secondary necrosis and inflammation^[72]^. The ECM, a key noncellular component to all organs and tissue, also contributes to atherosclerotic disease progression. Although there is not currently a lot known about the specific interplay between senescent cells and the ECM, proinflammatory factors in senescent cells can promote fibrotic changes, increasing collagen deposition in the ECM which is associated with the progression of atherogenesis^[73]^. Senescent cells can also degenerate the fibrous cap that usually prevents atherogenic plaque rupture, resulting in plaque instability and clinical consequences such as myocardial infarction and stroke. In studies using pharmacological or transgenic approaches to eliminate senescent cells in the Ldlr^−/−^ mouse model of atherosclerosis, the removal of senescent cells protected deteriorated fibrous caps in atherosclerotic lesions, reduced inflammation, and improved vascular function^[74]^. Other studies have shown that clearance of vascular cell senescence improved atherosclerotic progression along with other comorbidities in different mouse models of disease^[75–77]^. These findings indicate that there may be a therapeutic benefit of utilizing senolytic agents in atherosclerosis and reducing morbidity and mortality from CVD.

## SENOLYTICS

### Targeting senescent endothelial cells to improve vascular function

Senolytics are an emerging class of drugs able to selectively eliminate senescent cells through cell death mechanisms while leaving healthy, non-senescent cells alive. This concept was hypothesized after positive results were found in studies utilizing the INK-ATTAC mouse. In this model, Cdkn2a (encoding for p16Ink4a) is activated, inducing apoptosis in cells expressing p16Ink4a after treatment with the small molecule (AP20187)^[78]^. This model demonstrated that the removal of senescent cells improved health span and function in multiple tissues, setting the stage for the important work committed to identifying novel mechanisms and compounds that can selectively ablate senescent cells.

One such endeavor involved the use of another genetic model to identify/track senescent cells. To do so, Demaria *et al*. utilized the 3MR (trimodility reporter) fusion protein mouse and a mouse expressing a senescence-sensitive promoter, i.e., the tumor suppressor p16INK4a^[79]^. These investigators engineered a bacterial artificial chromosome (BAC) containing the murine p16INK4a locus, driving the 3MR expression. The result was a mouse model that was capable of tracking senescent cells via luminescence, SA-gal staining, and p16INK4a mRNA levels. Importantly, when cellular apoptosis was activated in these p16–3MR mice by exogenous ganciclovir (GCV), p16ink4a senescent cells were eliminated, alleviating atherogenesis and stabilizing plaques in the arterial wall^[79]^. Models such as this and the INK-ATTAC mouse model, an inducible model for elimination of p16Ink4a positive senescent cells, have provided proof of concept evidence that clearance of senescent cells can improve physiological function and reverse disease, paving the way for the exploration of senescent cells as potential pharmacological, therapeutic targets.

There are various pathways that may be manipulated to eliminate senescent cells by targeting anti-apoptotic mechanisms, termed senescent cell anti-apoptotic pathways (SCAPs). Because SCAPs are necessary for senescent cell viability, senescent survival proteins have been identified and tested through knockdown experiments, leading to the death of senescent but not non-senescent cells^[20]^. Drugs that have been developed to target SCAPs have seen the most promising results. A variety of senolytic drugs have been identified, but only a few have been researched in CVD. The combination treatment of dasatinib (D), a tyrosine kinase inhibitor, with quercetin (Q), a flavonoid and antioxidant, has proven highly effective in inducing apoptosis in senescent cells *in vitro* across multiple cell types, as well as in multiple animal models. D&Q treatment in human umbilical vein endothelial cells (HUVECs) demonstrated a senolytic effect, alleviating senescence via the inhibition of autocrine and paracrine actions of the SASP^[80]^. D&Q treatment in nonhuman primates also reduced SASP factors, increased immune function, and improved intestinal barrier function^[81]^. Zhu *et al*. first established that the pharmacological elimination of senescent cells with a single dose of D&Q could improve vascular endothelial function in aged mice^[20]^. Chronic senolytic treatment of D&Q in aged mice improved age-related vascular phenotypes such as vasomotor function, increased NO bioavailability, decreased aortic calcification and osteogenic signaling, and reduced chronic hypercholesterolemia^[82]^. These data indicate that D&Q may be a viable therapeutic intervention for age-related diseases. Presently, D&Q is one of the few senolytic drugs to be studied in a clinical trial setting. Hickson *et al*. found that in patients with diabetic kidney disease, treatment with D&Q reduced adipose tissue and epidermal senescent cell burden by demonstrating a reduction in p16^INK4A^ and p21^CIP1^-expressing cells, a reduced percentage of cells positive for senescence-associated -gal activity in adipocytes and epidermal cells, and a reduction in circulating SASP factors^[83]^. Quercetin alone has also been researched in numerous human and mouse studies of cardiovascular disease and demonstrated improvements in hypertension, hypolipidemia, hypoglycemia, and atherosclerosis, although measures of senescence were not included^[84]^. Thus, the combination of D&Q shows great promise as a senolytic treatment, and studying D&Q in the context of cardiovascular disease, both in long-term and short-term interventions, should be further explored.

The BCL-2 family of proteins is acknowledged as a pro-survival regulator of senescence that is also being explored as potential senolytics. These include ABT263 and Navitoclax, which are specific inhibitors of BCL-2 that lead to the induction of apoptosis in senescent cells^[22]^. Cell culture-based studies have demonstrated that Navitoclax reduces the viability of senescent HUVECs *in vitro* and treatment of cultured cells with BCL-xl siRNA also produces senolytic effects in HUVECs compared to other cell types^[22]^, suggesting EC specificity of targeting this pathway. The efficacy and hematological toxicity of the recently described BCL-2 inhibitors, fisetin, A1331852, and A1155463, are still being evaluated^[24]^. In a study in which aged p16–3MR mice were treated with GCV, inducing genetic clearance of senescent cells, or the senolytic ABT263, Clayton *et al*. found that GCV and ABT263 improved endothelial function via increased nitric oxide bioavailability and reduced oxidative stress^[85]^. In addition, arterial stiffness, measured by pulse wave velocity, was reduced to young levels in old mice treated with GCV and ABT263^[85]^. Additionally, a reduction in circulating SASP factors in concert with NO signaling was associated with enhanced NO-mediated EDD subsequent to the removal of senescent cells in both treatment groups^[85]^. Together, the available evidence suggests that cellular senescence and SASP play a role in vascular aging and that senolytic drugs show potential for ameliorating age-related vascular function. Furthermore, all identified BCL-2 inhibitors have been shown to have specific senoltyic effects in ECs compared to other cell types such as preadipocytes and VSMC, selectively eliminating senescent ECs while sparing proliferating ECs^[22]^. Although more studies investigating BLC-2 inhibitors on other vascular cell types must be performed, the beneficial impact of these EC-targeted senolytics on broad measures of arterial function suggests that endothelial cells are a good target for senolytic treatment.

A more recently defined form of non-apoptotic cell death, ferroptosis, has also been shown to have senolytic potential. Ferroptosis is a form of regulated cell death that relies on iron and is activated by the failure of glutathione-dependent antioxidant defense, resulting in unchecked lipid peroxidation and ultimately resulting in cell death^[86]^. Glutathione peroxidase 4 (GPX4) is an antioxidant selenoenzyme that can inhibit ferroptosis by quenching lipid peroxidation. Thus, GPX4 plays a vital role in the regulation of the ferroptosis pathway. RSL3 (RAS-selective lethal 3) is a small molecule inhibitor of GPX4 that has demonstrated the ability to induce ferroptotic cell death *in vitro*. Therapeutic effects of ferroptosis inducers (FINS) have received attention in cancer research^[87]^, but information on their role in the context of aging and senescence is deficient. Multiple studies have suggested the possibility of using FINs to selectively eliminate senescence, by demonstrating increased susceptibility to ferroptosis in senescent cells^[88,89]^. In a model of senescent tubular cells, treatment with RSL3 cleared senescent cells through ferroptosis and an *in vivo* study led to improved organ transplantation of aged mouse kidney^[90]^. While RSL3 may have off-target effects and is not stable in plasma, various GPX4 inhibitors have been developed and have demonstrated increased specificity to senescent cells *in vitro*^[91]^. Senescent cells have been reported to show ferroptosis resistance due to increased expression of GPX4, impaired ferritinophagy, upregulated iron uptake, and inactivated iron efflux via ferroportin^[89]^. However, because of senescent cell’s high iron content, they are good candidates for this form of cell death. Drugs that exploit senescent cell’s high iron pool such as TRX-CBI could be a safe and effective senolytic approach. TRX-CBI is a tumor-activated prodrug comprised of a trioxolane-based [TRX] sensor of Fe (II) conjugated to a cytotoxic cyclopropylbenzindoline [CBI] payload which demonstrated selective toxicity in Fe (II)-rich cancer cells^[92]^. In a study that treated primary human endothelial cells with TRX-CBI and FIN56 to induce ferroptosis, senescent cells were selectively eliminated^[42]^. Thus, ferroptosis may be a novel senolytic pathway that may be utilized to reduce senescent cell accumulation and improve endothelial and vascular function, although this requires further elucidation.

Recent research has also identified that cardiac glycosides (CGs), a group of steroids, exhibit senolytic properties. These compounds bind to and inhibit the plasma membrane Na^+^, K^+^ ATPase with high selectivity and affinity. The Na^+^, K^+^ ATPase regulates plasma membrane (PM) potential, and research has shown that inhibition of the subunit ATP1A1 of Na^+^, K^+^ ATPase enhances PM depolarization in senescent cells, which is necessary for senolysis. *In vivo* experiments screening different drugs in models of oncogene-induced senescence, conducted by Triana-Martinez *et al*. and Guerrero *et al*., demonstrated CGs to be a novel class of broad-spectrum senolytics^[93,94]^. Two identified CGs, digoxin^[95]^ and ouabain^[96]^, reduced senescent cell accumulation, immune infiltration, and inflammation both *in vitro* and *in vivo*. Ouabain and digoxin may induce increases in genetic expression of the proapoptotic BCL-2 family such as NOXA through activation of the JNK, GSK-3, and P38 pathways, partially mediating their senolytic effects, although this complete mechanism remains elusive^[94]^. The senolytic activity of CGs also indicates beneficial effects on atherosclerosis, pulmonary fibrosis, and anticancer therapies. Several clinical trials involving CGs include promising candidates as a therapeutic application in the context of cancer and aging^[97]^. However, the long-term effects of this class of senolytics require further study, as oubain may be associated with potential long-term deleterious effects. Thus, further research and clinical trials are necessary to evaluate the safety and efficacy of this class of senolytic in the treatment of other age-related diseases.

Although the elimination of senescent cells has shown promising results in cardiovascular and age-related diseases, examination of potential off-target or unanticipated side effects of senolytic therapy is also needed. Indeed, although a study examining pulmonary arterial hypertension (PAH) demonstrated high lung p16, p21, and γ-H2AX protein levels, as well as increased vascular senescence and DNA damage in patients with PAH, a detrimental effect of senolytic treatment has been described in the pulmonary circulation. For example, in a mouse model of hypoxia, eliminating senescent cells via the senolytics ABT263 or FOXO4-DRI aggravated the severity of monocrotaline-induced pulmonary hypertension^[98]^. This leads to the possibility that senolytics interventions may worsen pulmonary hemodynamics and strategies targeted at the elimination of senescent cells should consider the potential impact of senescence on the pulmonary system. More studies are necessary to better elucidate pulmonary senescence and the possibility of senescence as a protective mechanism against pulmonary hypertension and progression.

## SUMMARY AND FUTURE DIRECTIONS

This review aimed to provide a brief summary of the effects of aging on cardiovascular disease through the accumulation of senescence, highlighting the crucial involvement of vascular cells in the progression of atherosclerosis and other CVDs. We also sought to describe the potential of senolytics to improve vascular function and reduce CVD in aging. Endothelial dysfunction occurs with aging and promotes reductions in NO, increases in ROS, a proinflammatory phenotype, and is associated with an increase in senescent cell accumulation. Understanding how EC senescence influences endothelial dysfunction, atherosclerosis, and CVD is important in the identification/design of novel effective therapeutics. EC senescence is recognized as a contributing factor to endothelial dysfunction and is a major step in the development of atherosclerosis and other CVDs. Evidence suggests that genetic or pharmacological elimination of senescence, specifically in ECs, can attenuate vascular dysfunction and disease in aging through a reduction in the milieu of SASP factors present in senescence. These findings have also improved our understanding of the endothelium’s response to aging and how to combat endothelial dysfunction in this setting. Specifically, targeting endothelial cell senescence appears to be a promising strategy for maintaining endothelial functions and improving vascular health.

Preclinical evidence has shown the potential of senolytics for the treatment and prevention of CVD, leading to the investigation of senolytic therapy in the clinical setting. In a preliminary clinical trial investigating the effectiveness of senolytics, a treatment regimen involving a 3-day administration of D&Q per week for 3 weeks was implemented. This study was conducted on 14 patients with idiopathic pulmonary fibrosis and demonstrated high retention and completion rates, indicating the safety of this treatment^[99]^. Although there were no significant improvements in pulmonary function, treatment with D&Q led to notable improvements in physical function, as evidenced by increases in 6-min walk distance and 4-min gait speed. Moreover, correlations were observed between enhanced physical function and a reduction in SASP factors^[99]^. Additionally, preliminary data from a phase 1 clinical trial involving patients with diabetic kidney disease revealed that senolytic therapy with D&Q reduced senescence burden in both adipose and epidermis tissue. This reduction was associated with a decrease in circulating cytokines and matrix metalloproteinases^[83]^. In a 12-week pilot study of D&Q treatment on individuals with early-stage Alzheimer’s disease (AD), there was no significant difference between neuroimaging endpoints and cognitive function. However, results indicated a reduction in cytokine and chemokine levels associated with senescence as well as a trending increase in A42, a biomarker that is inversely related to Alzheimer’s disease diagnosis^[100]^. These studies show promise for the therapeutic potential of senolytic therapy in eliminating senescence, relieving SASP accumulation, and reducing inflammation, although more compounds with appropriate safety, tolerability, and feasibility need to be developed and investigated. Moreover, clinical trials using senolytic therapy in the context of atherosclerosis and cardiovascular disease should be conducted.

Currently, there is not enough research on the use and treatment of senolytic therapy in cardiovascular diseases in the clinical setting. It is unclear if senescent cell clearance, either systemically or in a cell-specific manner, will impact the cardiovascular system, specifically on health span in general. Furthermore, the long-term effects of senescent cell elimination, both systemic and tissue-specific, are not well known. More research must be conducted to answer these questions. While short-term clearance of senescent endothelial and vascular smooth muscle cells improved cardiovascular function and atherosclerosis in preclinical models, further studies are necessary to ensure that the elimination of this cell population has no adverse effects on systemic function, both long-term and short-term. Nevertheless, the potential of senolytics to transform age-related cardiovascular diseases and improve health span is an exciting frontier.

## Data Availability

Data and articles included in this review are available on PUBMED.

## References

[1] Virani SS, Alonso A, Benjamin EJ, Heart disease and stroke statistics-2020 update: a report from the American heart association. Circulation 2020;141:e139–596. 31992061 10.1161/CIR.0000000000000757

[2] Majnarić L, Bosnić Z, Kurevija T, Wittlinger T. Cardiovascular risk and aging: the need for a more comprehensive understanding. J Geriatr Cardiol 2021;18:462–78. 34220975 10.11909/j.issn.1671-5411.2021.06.004 PMC8220387

[3] Meijs MF, de Windt LJ, de Jonge N, Left ventricular hypertrophy: a shift in paradigm. Curr Med Chem 2007;14:157–71. 17266575 10.2174/092986707779313354

[4] Mitchell GF, Parise H, Benjamin EJ, Changes in arterial stiffness and wave reflection with advancing age in healthy men and women: the framingham heart study. Hypertension 2004;43:1239–45. 15123572 10.1161/01.HYP.0000128420.01881.aa

[5] Schulman SP, Lakatta EG, Fleg JL, Lakatta L, Becker LC, Gerstenblith G. Age-related decline in left ventricular filling at rest and exercise. Am J Physiol 1992;263:H1932–8. 1362335 10.1152/ajpheart.1992.263.6.H1932

[6] Izzo JL Jr, Shykoff BE. Arterial stiffness: clinical relevance, measurement, and treatment. Rev Cardiovasc Med 2001;2:29–40. 12478235

[7] Liberale L, Camici GG. The role of vascular aging in atherosclerotic plaque development and vulnerability. Curr Pharm Des 2019;25:3098–111. 31470777 10.2174/1381612825666190830175424

[8] Lakatta EG, Levy D. Arterial and cardiac aging: major shareholders in cardiovascular disease enterprises: Part I: aging arteries: a “set up” for vascular disease. Circulation 2003;107:139–46. 12515756 10.1161/01.cir.0000048892.83521.58

[9] Pinto AR, Ilinykh A, Ivey MJ, Revisiting cardiac cellular composition. Circ Res 2016;118:400–9. 26635390 10.1161/CIRCRESAHA.115.307778 PMC4744092

[10] Brutsaert DL. Cardiac endothelial-myocardial signaling: its role in cardiac growth, contractile performance, and rhythmicity. Physiol Rev 2003;83:59–115. 12506127 10.1152/physrev.00017.2002

[11] Minamino T, Miyauchi H, Yoshida T, Ishida Y, Yoshida H, Komuro I. Endothelial cell senescence in human atherosclerosis: role of telomere in endothelial dysfunction. Circulation 2002;105:1541–4. 11927518 10.1161/01.cir.0000013836.85741.17

[12] Donato AJ, Machin DR, Lesniewski LA. Mechanisms of dysfunction in the aging vasculature and role in age-related disease. Circ Res 2018;123:825–48. 30355078 10.1161/CIRCRESAHA.118.312563 PMC6207260

[13] Gorgoulis V, Adams PD, Alimonti A, Cellular senescence: defining a path forward. Cell 2019;179:813–27. 31675495 10.1016/j.cell.2019.10.005

[14] Campisi J Senescent cells, tumor suppression, and organismal aging: good citizens, bad neighbors. Cell 2005;120:513–22. 15734683 10.1016/j.cell.2005.02.003

[15] Beauséjour CM, Krtolica A, Galimi F, Reversal of human cellular senescence: roles of the p53 and p16 pathways. EMBO J 2003;22:4212–22. 12912919 10.1093/emboj/cdg417 PMC175806

[16] Sapieha P, Mallette FA. Cellular senescence in postmitotic cells: beyond growth arrest. Trends Cell Biol 2018;28:595–607. 29704982 10.1016/j.tcb.2018.03.003

[17] Englund DA, Jolliffe AM, Hanson GJ, Senotherapeutic drug treatment ameliorates chemotherapy-induced cachexia. JCI Insight 2024;9:e169512. 38051584 10.1172/jci.insight.169512 PMC10906225

[18] Hayflick L, Moorhead PS. The serial cultivation of human diploid cell strains. Exp Cell Res 1961;25:585–621. 13905658 10.1016/0014-4827(61)90192-6

[19] Schafer MJ, Zhang X, Kumar A, The senescence-associated secretome as an indicator of age and medical risk. JCI Insight 2020;5:133668. 32554926 10.1172/jci.insight.133668 PMC7406245

[20] Zhu Y, Tchkonia T, Pirtskhalava T, The achilles’ heel of senescent cells: from transcriptome to senolytic drugs. Aging Cell 2015;14:644–58. 25754370 10.1111/acel.12344 PMC4531078

[21] Chen LS, Balakrishnan K, Gandhi V. Inflammation and survival pathways: chronic lymphocytic leukemia as a model system. Biochem Pharmacol 2010;80:1936–45. 20696142 10.1016/j.bcp.2010.07.039 PMC3749885

[22] Zhu Y, Tchkonia T, Fuhrmann-Stroissnigg H, Identification of a novel senolytic agent, navitoclax, targeting the Bcl-2 family of anti-apoptotic factors. Aging Cell 2016;15:428–35. 26711051 10.1111/acel.12445 PMC4854923

[23] Chaib S, Tchkonia T, Kirkland JL. Cellular senescence and senolytics: the path to the clinic. Nat Med 2022;28:1556–68. 35953721 10.1038/s41591-022-01923-y PMC9599677

[24] Zhu Y, Doornebal EJ, Pirtskhalava T, New agents that target senescent cells: the flavone, fisetin, and the BCL-XL inhibitors, A1331852 and A1155463. Aging 2017;9:955–63. 28273655 10.18632/aging.101202 PMC5391241

[25] Hernandez-Segura A, de Jong TV, Melov S, Guryev V, Campisi J, Demaria M. Unmasking transcriptional heterogeneity in senescent cells. Curr Biol 2017;27:2652–60.e4. 28844647 10.1016/j.cub.2017.07.033 PMC5788810

[26] d’Adda di Fagagna F, Reaper PM, Clay-Farrace L, A DNA damage checkpoint response in telomere-initiated senescence. Nature 2003;426:194–8. 14608368 10.1038/nature02118

[27] Herbig U, Jobling WA, Chen BP, Chen DJ, Sedivy JM. Telomere shortening triggers senescence of human cells through a pathway involving ATM, p53, and p21(CIP1), but not p16(INK4a). Mol Cell 2004;14:501–13. 15149599 10.1016/s1097-2765(04)00256-4

[28] Burgering BM, Medema RH. Decisions on life and death: FOXO forkhead transcription factors are in command when PKB/Akt is off duty. J Leukoc Biol 2003;73:689–701. 12773501 10.1189/jlb.1202629

[29] Di Micco R, Fumagalli M, Cicalese A, Oncogene-induced senescence is a DNA damage response triggered by DNA hyper-replication. Nature 2006;444:638–42. 17136094 10.1038/nature05327

[30] Baar MP, Brandt RMC, Putavet DA, Targeted apoptosis of senescent cells restores tissue homeostasis in response to chemotoxicity and aging. Cell 2017;169:132–47.e16. 28340339 10.1016/j.cell.2017.02.031 PMC5556182

[31] Campisi J, d’Adda di Fagagna F. Cellular senescence: when bad things happen to good cells. Nat Rev Mol Cell Biol 2007;8:729–40. 17667954 10.1038/nrm2233

[32] Kirkland JL, Tchkonia T, Zhu Y, Niedernhofer LJ, Robbins PD. The clinical potential of senolytic drugs. J Am Geriatr Soc 2017;65:2297–301. 28869295 10.1111/jgs.14969 PMC5641223

[33] Acosta JC, Banito A, Wuestefeld T, A complex secretory program orchestrated by the inflammasome controls paracrine senescence. Nat Cell Biol 2013;15:978–90. 23770676 10.1038/ncb2784 PMC3732483

[34] Xu M, Pirtskhalava T, Farr JN, Senolytics improve physical function and increase lifespan in old age. Nat Med 2018;24:1246–56. 29988130 10.1038/s41591-018-0092-9 PMC6082705

[35] Freund A, Patil CK, Campisi J. p38MAPK is a novel DNA damage response-independent regulator of the senescence-associated secretory phenotype. EMBO J 2011;30:1536–48. 21399611 10.1038/emboj.2011.69 PMC3102277

[36] Cao X, Li M. A new pathway for senescence regulation. Genom Proteom Bioinf 2015;13:333–5. 10.1016/j.gpb.2015.11.002 PMC4747646 26777575

[37] Sung JY, Lee KY, Kim JR, Choi HC. Interaction between mTOR pathway inhibition and autophagy induction attenuates adriamyc-ininduced vascular smooth muscle cell senescence through decreased expressions of p53/p21/p16. Exp Gerontol 2018;109:51–8. 28797827 10.1016/j.exger.2017.08.001

[38] Sun L, Wu J, Du F, Chen X, Chen ZJ. Cyclic GMP-AMP synthase is a cytosolic DNA sensor that activates the type I interferon pathway. Science 2013;339:786–91. 23258413 10.1126/science.1232458 PMC3863629

[39] Sakai C, Ueda K, Goda K, A possible role for proinflammatory activation via cGAS-STING pathway in atherosclerosis induced by accumulation of DNA double-strand breaks. Sci Rep 2023;13:16470. 37777633 10.1038/s41598-023-43848-7 PMC10542807

[40] Luo W, Zou X, Wang Y, Critical role of the cGAS-STING pathway in doxorubicin-induced cardiotoxicity. Circ Res 2023;132:e223–42. 37154056 10.1161/CIRCRESAHA.122.321587

[41] Hoare M, Ito Y, Kang TW, NOTCH1 mediates a switch between two distinct secretomes during senescence. Nat Cell Biol 2016;18:979–92. 27525720 10.1038/ncb3397 PMC5008465

[42] Admasu TD, Kim K, Rae M, Selective ablation of primary and paracrine senescent cells by targeting iron dyshomeostasis. Cell Rep 2023;42:112058. 36753419 10.1016/j.celrep.2023.112058

[43] Wang TW, Johmura Y, Suzuki N, Blocking PD-L1-PD-1 improves senescence surveillance and ageing phenotypes. Nature 2022;611:358–64. 36323784 10.1038/s41586-022-05388-4

[44] Amor C, Feucht J, Leibold J, Senolytic CAR T cells reverse senescence-associated pathologies. Nature 2020;583:127–32. 32555459 10.1038/s41586-020-2403-9 PMC7583560

[45] Suda M, Shimizu I, Katsuumi G, Senolytic vaccination improves normal and pathological age-related phenotypes and increases lifespan in progeroid mice. Nat Aging 2021;1:1117–26. 37117524 10.1038/s43587-021-00151-2

[46] Yoshida S, Nakagami H, Hayashi H, The CD153 vaccine is a senotherapeutic option for preventing the accumulation of senescent T cells in mice. Nat Commun 2020;11:2482. 32424156 10.1038/s41467-020-16347-w PMC7235045

[47] Zhu Y, Prata LGPL, Gerdes EOW, Orally-active, clinically-translatable senolytics restore α-Klotho in mice and humans. EBioMedicine 2022;77:103912. 35292270 10.1016/j.ebiom.2022.103912 PMC9034457

[48] Passos JF, Saretzki G, Ahmed S, Mitochondrial dysfunction accounts for the stochastic heterogeneity in telomere-dependent senescence. PLoS Biol 2007;5:e110. 17472436 10.1371/journal.pbio.0050110 PMC1858712

[49] Kirkman DL, Robinson AT, Rossman MJ, Seals DR, Edwards DG. Mitochondrial contributions to vascular endothelial dysfunction, arterial stiffness, and cardiovascular diseases. Am J Physiol Heart Circ Physiol 2021;320:H2080–100. 33834868 10.1152/ajpheart.00917.2020 PMC8163660

[50] von Zglinicki T Oxidative stress shortens telomeres. Trends Biochem Sci 2002;27:339–44. 12114022 10.1016/s0968-0004(02)02110-2

[51] Lafargue A, Degorre C, Corre I, Ionizing radiation induces long-term senescence in endothelial cells through mitochondrial respiratory complex II dysfunction and superoxide generation. Free Radic Biol Med 2017;108:750–9. 28431961 10.1016/j.freeradbiomed.2017.04.019

[52] Miwa S, Kashyap S, Chini E, von Zglinicki T. Mitochondrial dysfunction in cell senescence and aging. J Clin Invest 2022;132:e158447. 35775483 10.1172/JCI158447 PMC9246372

[53] Lee S, Jeong SY, Lim WC, Mitochondrial fission and fusion mediators, hFis1 and OPA1, modulate cellular senescence. J Biol Chem 2007;282:22977–83. 17545159 10.1074/jbc.M700679200

[54] Gioscia-Ryan RA, Battson ML, Cuevas LM, Eng JS, Murphy MP, Seals DR. Mitochondria-targeted antioxidant therapy with MitoQ ameliorates aortic stiffening in old mice. J Appl Physiol 2018;124:1194–202. 29074712 10.1152/japplphysiol.00670.2017 PMC6008077

[55] Zhong L, Deng J, Gu C, Protective effect of MitoQ on oxidative stress-mediated senescence of canine bone marrow mesenchymal stem cells via activation of the Nrf2/ARE pathway. In Vitro Cell Dev Biol Anim 2021;57:685–94. 34518994 10.1007/s11626-021-00605-2

[56] Saretzki G, Murphy MP, von Zglinicki T. MitoQ counteracts telomere shortening and elongates lifespan of fibroblasts under mild oxidative stress. Aging Cell 2003;2:141–3. 12882327 10.1046/j.1474-9728.2003.00040.x

[57] Wu M, Rementer C, Giachelli CM. Vascular calcification: an update on mechanisms and challenges in treatment. Calcif Tissue Int 2013;93:365–73. 23456027 10.1007/s00223-013-9712-z PMC3714357

[58] Bu LL, Yuan HH, Xie LL, Guo MH, Liao DF, Zheng XL. New dawn for atherosclerosis: vascular endothelial cell senescence and death. Int J Mol Sci 2023;24:15160. 37894840 10.3390/ijms242015160 PMC10606899

[59] McHugh D, Gil J. Senescence and aging: causes, consequences, and therapeutic avenues. J Cell Biol 2018;217:65–77. 29114066 10.1083/jcb.201708092 PMC5748990

[60] Rossman MJ, Kaplon RE, Hill SD, Endothelial cell senescence with aging in healthy humans: prevention by habitual exercise and relation to vascular endothelial function. Am J Physiol Heart Circ Physiol 2017;313:H890–5. 28971843 10.1152/ajpheart.00416.2017 PMC5792201

[61] Bloom SI, Islam MT, Lesniewski LA, Donato AJ. Mechanisms and consequences of endothelial cell senescence. Nat Rev Cardiol 2023;20:38–51. 35853997 10.1038/s41569-022-00739-0 PMC10026597

[62] Jia G, Aroor AR, Jia C, Sowers JR. Endothelial cell senescence in aging-related vascular dysfunction. Biochim Biophys Acta Mol Basis Dis 2019;1865:1802–9. 31109450 10.1016/j.bbadis.2018.08.008

[63] Bloom SI, Liu Y, Tucker JR, Endothelial cell telomere dysfunction induces senescence and results in vascular and metabolic impairments. Aging Cell 2023;22:e13875. 37259606 10.1111/acel.13875 PMC10410008

[64] Sato I, Morita I, Kaji K, Ikeda M, Nagao M, Murota S. Reduction of nitric oxide producing activity associated with in vitro aging in cultured human umbilical vein endothelial cell. Biochem Biophys Res Commun 1993;195:1070–6. 7690550 10.1006/bbrc.1993.2153

[65] Yokoyama M, Shimizu I, Nagasawa A, p53 plays a crucial role in endothelial dysfunction associated with hyperglycemia and ischemia. J Mol Cell Cardiol 2019;129:105–17. 30790589 10.1016/j.yjmcc.2019.02.010

[66] Freeman BD, Machado FS, Tanowitz HB, Desruisseaux MS. Endothelin-1 and its role in the pathogenesis of infectious diseases. Life Sci 2014;118:110–9. 24780317 10.1016/j.lfs.2014.04.021 PMC4538933

[67] Uryga AK, Bennett MR. Ageing induced vascular smooth muscle cell senescence in atherosclerosis. J Physiol 2016;594:2115–24. 26174609 10.1113/JP270923 PMC4933105

[68] Gimbrone MA Jr, García-Cardeña G. Endothelial cell dysfunction and the pathobiology of atherosclerosis. Circ Res 2016;118:620–36. 26892962 10.1161/CIRCRESAHA.115.306301 PMC4762052

[69] Nishiguchi T, Tanaka A, Taruya A, Local matrix metalloproteinase 9 level determines early clinical presentation of ST-segment-elevation myocardial infarction. Arterioscler Thromb Vasc Biol 2016;36:2460–7. 27687605 10.1161/ATVBAHA.116.308099

[70] Childs BG, Baker DJ, Wijshake T, Conover CA, Campisi J, van Deursen JM. Senescent intimal foam cells are deleterious at all stages of atherosclerosis. Science 2016;354:472–7. 27789842 10.1126/science.aaf6659 PMC5112585

[71] Owens GK, Kumar MS, Wamhoff BR. Molecular regulation of vascular smooth muscle cell differentiation in development and disease. Physiol Rev 2004;84:767–801. 15269336 10.1152/physrev.00041.2003

[72] Grootaert MOJ, Moulis M, Roth L, Vascular smooth muscle cell death, autophagy and senescence in atherosclerosis. Cardiovasc Res 2018;114:622–34. 29360955 10.1093/cvr/cvy007

[73] Levi N, Papismadov N, Solomonov I, Sagi I, Krizhanovsky V. The ECM path of senescence in aging: components and modifiers. FEBS J 2020;287:2636–46. 32145148 10.1111/febs.15282

[74] Childs BG, Zhang C, Shuja F, Senescent cells suppress innate smooth muscle cell repair functions in atherosclerosis. Nat Aging 2021;1:698–714. 34746803 10.1038/s43587-021-00089-5 PMC8570576

[75] Lazaro I, Oguiza A, Recio C, Targeting HSP90 ameliorates nephropathy and atherosclerosis through suppression of NF-κB and STAT signaling pathways in diabetic mice. Diabetes 2015;64:3600–13. 26116697 10.2337/db14-1926

[76] Johmura Y, Yamanaka T, Omori S, Senolysis by glutaminolysis inhibition ameliorates various age-associated disorders. Science 2021;371:265–70. 33446552 10.1126/science.abb5916

[77] Garrido AM, Kaistha A, Uryga AK, Efficacy and limitations of senolysis in atherosclerosis. Cardiovasc Res 2022;118:1713–27. 34142149 10.1093/cvr/cvab208 PMC9215197

[78] Baker DJ, Wijshake T, Tchkonia T, Clearance of p16Ink4a-positive senescent cells delays ageing-associated disorders. Nature 2011;479:232–6. 22048312 10.1038/nature10600 PMC3468323

[79] Demaria M, Ohtani N, Youssef SA, An essential role for senescent cells in optimal wound healing through secretion of PDGF-AA. Dev Cell 2014;31:722–33. 25499914 10.1016/j.devcel.2014.11.012 PMC4349629

[80] Fan T, Du Y, Zhang M, Zhu AR, Zhang J. Senolytics cocktail dasatinib and quercetin alleviate human umbilical vein endothelial cell senescence via the TRAF6-MAPK-NF-κB axis in a YTHDF2-dependent manner. Gerontology 2022;68:920–34. 35468611 10.1159/000522656

[81] Ruggiero AD, Vemuri R, Blawas M, Long-term dasatinib plus quercetin effects on aging outcomes and inflammation in nonhuman primates: implications for senolytic clinical trial design. Geroscience 2023;45:2785–803. 37261678 10.1007/s11357-023-00830-5 PMC10643765

[82] Roos CM, Zhang B, Palmer AK, Chronic senolytic treatment alleviates established vasomotor dysfunction in aged or atherosclerotic mice. Aging Cell 2016;15:973–7. 26864908 10.1111/acel.12458 PMC5013022

[83] Hickson LJ, Langhi Prata LGP, Bobart SA, Senolytics decrease senescent cells in humans: preliminary report from a clinical trial of dasatinib plus quercetin in individuals with diabetic kidney disease. EBioMedicine 2019;47:446–56. 31542391 10.1016/j.ebiom.2019.08.069 PMC6796530

[84] Papakyriakopoulou P, Velidakis N, Khattab E, Valsami G, Korakianitis I, Kadoglou NP. Potential pharmaceutical applications of quercetin in cardiovascular diseases. Pharmaceuticals 2022;15:1019. 36015169 10.3390/ph15081019 PMC9412669

[85] Clayton ZS, Rossman MJ, Mahoney SA, Cellular senescence contributes to large elastic artery stiffening and endothelial dysfunction with aging: amelioration with senolytic treatment. Hypertension 2023;80:2072–87. 37593877 10.1161/HYPERTENSIONAHA.123.21392 PMC10530538

[86] Stockwell BR, Friedmann Angeli JP, Bayir H, Ferroptosis: a regulated cell death nexus linking metabolism, redox biology, and disease. Cell 2017;171:273–85. 28985560 10.1016/j.cell.2017.09.021 PMC5685180

[87] Hassannia B, Vandenabeele P, Vanden Berghe T. Targeting ferroptosis to iron out cancer. Cancer Cell 2019;35:830–49. 31105042 10.1016/j.ccell.2019.04.002

[88] Wei Z, Hao C, Huangfu J, Srinivasagan R, Zhang X, Fan X. Aging lens epithelium is susceptible to ferroptosis. Free Radic Biol Med 2021;167:94–108. 33722625 10.1016/j.freeradbiomed.2021.02.010 PMC8096685

[89] Go S, Kang M, Kwon SP, Jung M, Jeon OH, Kim BS. The senolytic drug JQ1 removes senescent cells via ferroptosis. Tissue Eng Regen Med 2021;18:841–50. 34003467 10.1007/s13770-021-00346-z PMC8440740

[90] Liao CM, Wulfmeyer VC, Chen R, Induction of ferroptosis selectively eliminates senescent tubular cells. Am J Transplant 2022;22:2158–68. 35607817 10.1111/ajt.17102

[91] Du Y, Guo Z. Recent progress in ferroptosis: inducers and inhibitors. Cell Death Discov 2022;8:501. 36581640 10.1038/s41420-022-01297-7 PMC9800531

[92] Spangler B, Fontaine SD, Shi Y, A novel tumor-activated prodrug strategy targeting ferrous iron is effective in multiple preclinical cancer models. J Med Chem 2016;59:11161–70. 27936709 10.1021/acs.jmedchem.6b01470 PMC5184369

[93] Triana-Martínez F, Picallos-Rabina P, Da Silva-Álvarez S, Identification and characterization of cardiac glycosides as senolytic compounds. Nat Commun 2019;10:4731. 31636264 10.1038/s41467-019-12888-x PMC6803708

[94] Guerrero A, Herranz N, Sun B, Cardiac glycosides are broad-spectrum senolytics. Nat Metab 2019;1:1074–88. 31799499 10.1038/s42255-019-0122-z PMC6887543

[95] Shi H, Mao X, Zhong Y, Digoxin reduces atherosclerosis in apolipoprotein E-deficient mice. Br J Pharmacol 2016;173:1517–28. 26879387 10.1111/bph.13453 PMC4831306

[96] Li B, Huang X, Liu Z, Ouabain ameliorates bleomycin induced pulmonary fibrosis by inhibiting proliferation and promoting apoptosis of lung fibroblasts. Am J Transl Res 2018;10:2967–74. PubMed 30323883 PMC6176221

[97] Tzekaki EE, Geromichalos G, Lavrentiadou SN, Tsantarliotou MP, Pantazaki AA, Papaspyropoulos A. Oleuropein is a natural inhibitor of PAI-1-mediated proliferation in human ER-/PR- breast cancer cells. Breast Cancer Res Treat 2021;186:305–16. 33389400 10.1007/s10549-020-06054-x

[98] Born E, Lipskaia L, Breau M, Eliminating senescent cells can promote pulmonary hypertension development and progression. Circulation 2023;147:650–66. 36515093 10.1161/CIRCULATIONAHA.122.058794

[99] Justice JN, Nambiar AM, Tchkonia T, Senolytics in idiopathic pulmonary fibrosis: results from a first-in-human, open-label, pilot study. EBioMedicine 2019;40:554–63. 30616998 10.1016/j.ebiom.2018.12.052 PMC6412088

[100] Gonzales MM, Garbarino VR, Kautz TF, Senolytic therapy in mild Alzheimer’s disease: a phase 1 feasibility trial. Nat Med 2023;29:2481–8. 37679434 10.1038/s41591-023-02543-w PMC10875739

